# Implementation of oral versus intravenous antibiotics in clinical practice at a specialized orthopedic infection unit: a descriptive retrospective cohort study

**DOI:** 10.2340/17453674.2025.44571

**Published:** 2025-09-11

**Authors:** Robin BAWER, Anton A N PETERLIN, Jakob BAK, Hans GOTTLIEB

**Affiliations:** 1 Department of Orthopedic Surgery at Copenhagen University Hospital, Herlev Hospital; 2 Department of Veterinary and Animal Sciences, University of Copenhagen, Experimental Pathology, Frederiksberg, Denmark

## Abstract

**Background and purpose:**

The Oral Versus Intravenous Antibiotics (OVIVA) trial demonstrated that oral antibiotic therapy was noninferior to intravenous antibiotic therapy when used during the initial 6 weeks in the treatment of bone and joint infections (BJIs). We aimed to evaluate clinical outcomes, antibiotic treatment details, and complication rates following the implementation of the OVIVA protocol.

**Methods:**

All patients treated for BJIs between September 2019 and September 2020 at the specialized orthopedic infection unit of Herlev Hospital were eligible for inclusion. This study included data on patient demographics, antibiotic regimens, type of infection, microbiology, length of stay, adverse drug reactions, and definite treatment failure at 1 year.

**Results:**

A cohort of 129 patients was included. After a median of 7 days of intravenous therapy, 127 patients underwent an early switch to oral antibiotics. The most frequently used class of oral antibiotics was penicillins (68%). Adverse drug reactions, mostly gastrointestinal, occurred in 36% of all patients. Definite treatment failure at 1 year was 13% with oral antibiotics.

**Conclusion:**

We found a comparably low failure rate of 13% among patients who were able to transition to oral antibiotics when applying the treatment protocol from the OVIVA study.

Traditionally, the treatment of bone and joint infections (BJIs) has relied on prolonged intravenous (IV) antibiotics and repeated surgery [1]. Earlier literature suggests that osteomyelitis is rarely controlled without complete surgical debridement and extended IV antibiotics [2,3]. For prosthetic joint infections (PJIs), even longer courses of IV and oral antibiotics are typically recommended [4-6].

The Oral Versus Intravenous Antibiotics (OVIVA) trial was the first prospective randomized clinical study to challenge the necessity of prolonged IV antibiotics in BJIs [7]. The primary outcome of the trial was definitive treatment failure within 1 year, and it showed noninferiority of oral antibiotics when used during the initial 6 weeks in patients randomized to receive either IV or oral antibiotics within 7 days after surgery. A change from IV to oral treatment may be an advantage to the patients and the healthcare system, if it is safe. Only a few have tried to confirm the OVIVA study, which is why there is a need for more evidence.

To the best of our knowledge, Azamgarhi et al. at the Royal National Orthopaedic Hospital (RNOH) conducted the only study assessing the reproducibility of the OVIVA trial findings [8]. Both the OVIVA and RNOH studies demonstrated that oral antibiotics were noninferior to IV therapy, establishing their efficacy while challenging the traditional reliance on IV therapy. Furthermore, secondary outcomes in the OVIVA study showed similar rates of serious adverse drug reactions (ADRs), and both studies associated IV therapy with longer hospital stays. Additionally, a systematic review by Haddad et al. also showed that oral antibiotic therapy is a safe and effective alternative to IV therapy in the treatment of BJIs [9].

Our study reports the clinical outcomes of patients treated for BJIs according to the OVIVA protocol. We aimed to evaluate the clinical outcome, antibiotic treatment details, and complication rates.

## Methods

In September 2019 the specialized orthopedic infection unit at Herlev University Hospital implemented the OVIVA protocol in the management of BJIs in conjunction with an already established standardized 1-stage surgical technique.

### Study design

This study is retrospective, and data were collected after the implementation of the OVIVA protocol. All patients treated for BJIs between September 2019 and September 2020 at the orthopedic infection unit of Herlev Hospital were eligible for inclusion in the study. 2-stage revisions of PJIs were performed exclusively at an affiliated hospital and therefore not included in this study. In contrast, cases managed with debridement and implant retention (DAIR) and 1-stage revisions were included. The study is reported according to the STROBE guidelines.

### Clinical setting

BJIs were defined as osteomyelitis (including fracture-related infections, chronic osteomyelitis, and diabetic foot osteomyelitis), as well as PJIs and septic arthritis. Barring any allergies or prior known bacteria identification, empirical IV antibiotics were penicillin G (1.2 g, 4 times daily) and cloxacillin (1 g, 4 times daily). This antibiotic regimen was based on recommendations from the clinical microbiologists at Herlev Hospital. Antibiotic guidance and duration were determined at weekly conferences held by a multidisciplinary team of microbiologists, infectious disease specialists, and orthopedic surgeons. In cases of culture-negative infection, the empirical antibiotic treatment was continued, which is common practice if the clinical signs suggest ongoing infection. Blood test monitoring was routinely performed during hospitalization. IV catheters were ultrasound-guided by nurses as either longlines/midlines. All patients had a minimum follow-up of 1 year, including regular visits with specialized nurses and outpatient consultations.

The 1-stage management of BJIs included the collection of 5 deep bone or tissue samples, thorough debridement, and removal of metalware when feasible. This was followed by saline irrigation and application of an antibiotic-eluting calcium sulfate/hydroxyapatite graft substitute (Cerament G: 17.5 mg gentamicin/mL; Cerament V: 66 mg vancomycin/mL; Bonesupport AB, Lund, Sweden). The procedure was concluded with primary wound closure, with or without assistance from plastic surgeons.

Patient demographics, ASA scores, type of infection, surgical intervention, length of stay (LOS), microbiological findings, ADRs, and failure/clinical outcome were included in this study, as well as details on antibiotic type, duration and transitions retrieved from electronic medical records.

### Primary outcome

Clinical outcome was based on definite treatment failure defined as the presence of at least 1 clinical criterion (draining sinus tract arising from bone or prosthesis), microbiological criterion (phenotypically indistinguishable bacteria isolates from 2 or more out of 5 deep-tissue samples or pathogenic organism from single closed aspirate or biopsy), or histologic criterion (presence of characteristic inflammatory infiltrate or microorganisms) in accordance with earlier studies’ protocol [7,8].

### Secondary outcomes

All reported ADRs during the first 6 weeks were presented according to earlier studies with regard to type and severity [8]. ADRs were categorized as gastrointestinal, neuromuscular, biochemical/hematological, immunological, or microbiological. Details regarding their management and hospitalizations related to ADRs or any other cause were noted. IV-line complications were reported as incidence per 1,000 line-days, and included mechanical problems, thromboembolism, or infection. LOS was calculated based on admission and discharge dates from hospital records. While LOS data prior to the implementation of OVIVA were not included in this study, the standard treatment protocol at the time involved at least 14 days of IV antibiotic therapy.

### Statistics

Outcome analysis excluded patients who died within the minimum 1-year follow-up period to ensure adequate time for assessing treatment failure. Statistical analysis was performed using IBM SPSS Statistics version 25 (IBM Corp, Armonk, NY, USA).

### Ethics, data sharing plan, funding, and disclosures

The study was approved as a quality control study by the local legal department at the hospital. Data is available by contacting the corresponding author upon reasonable request. The authors received no financial or material support for the research, authorship, and/or publication of this article. ICJME disclosure of interest forms are available as supplementary data on the article page, doi: 10.2340/17453674.2025.44571

## Results

Patient inclusion, results, and primary outcome data are illustrated in the Flowchart. 132 patients were treated for BJIs between September 2019 and September 2020 at Herlev Hospital. 3 patients were excluded as they did not consent to review of their electronic medical records but were still treated according to the OVIVA protocol. Consequently, 129 patients were included in total, and 127 patients were able to switch to oral therapy.

All patients underwent surgical treatment, except for 1 case of septic arthritis, which was managed nonoperatively. The most common type of BJI in patients treated with oral antibiotics was osteomyelitis, which included chronic osteomyelitis (15 patients), fracture-related infections (51 patients), and diabetic foot osteomyelitis (27 patients). This group accounted for 73%, followed by PJIs at 17% and septic arthritis at 9.4% (solely native knee joints). None of the patients included had any type of spinal infections. Patients treated solely with IV antibiotics had diabetes foot osteomyelitis (2 patients).

**Figure:**
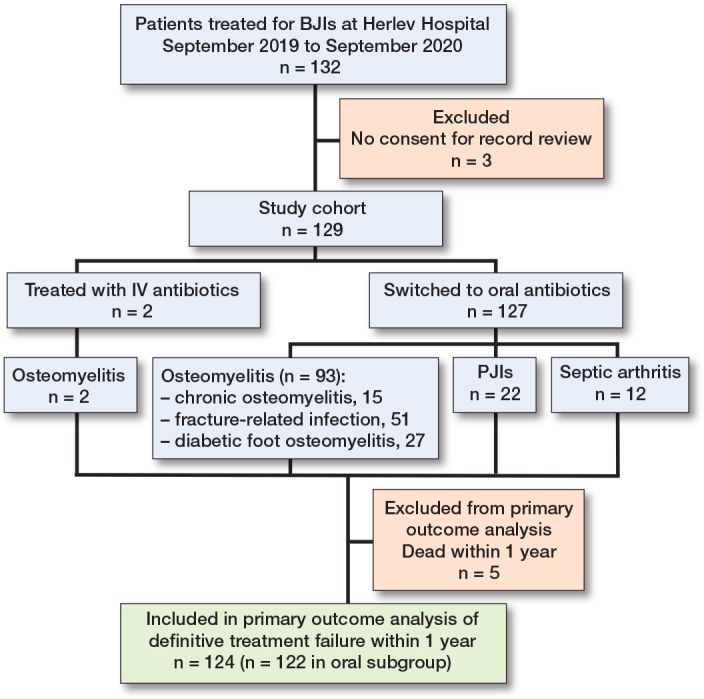
Flowchart visualizing patient selection, results, and primary outcome.BJI: bone and joint infection, PJI: prosthetic joint infection, IV: intravenous.

### Patient characteristics and treatment

Basic demographics and baseline characteristics showed that a larger proportion of patients was ASA ≥ 3, a smaller proportion was ASA 1, and a similar proportion was ASA 2 in the Herlev cohort compared with the RNOH cohort (Table 1).

**Table 1:** Baseline characteristics and indication for antibiotic therapy: comparison of Herlev and RNOH oral subgroups. Values are n (%) or median with (interquartile range)

Item	Herlev	RNOH
No. of patients treated	127	121
Median age (IQR)	66 (54–77)	63 (51–71)
Sex, male/female	71/56	64/57
ASA score		
1	17 (13)	41 (34)
2	58 (46)	63 (52)
≥3	52 (41)	16 (13)
Days of antibiotic therapy (IQR)	42 (42–50)	49 (42–84)
Days of IV antibiotic therapy (IQR)	7 (7–7)	
Prosthetic joint infections		
Debridement and implant retention	11 (8.7)	9 (7.4)
Implant removed	6 (4.7)	40 (33)
1-stage exchange	5 (3.9)	35 (29)
Osteomyelitis	93 (73)	27 (22)
Septic arthritis	12 (9.4)	0
Surgery for discitis, spinal osteomyelitis, or epidural abscess	0	9 (7.4)
Removal of other orthopedic device for infection	0	1 (0.8)

ASA: American Society of Anesthesiologists, IQR: interquartile range, IV: intravenous, RNOH: Royal National Orthopaedic Hospital.

PJIs (n = 22) were managed predominantly with DAIR in 11 cases, followed by a longer duration of antibiotic treatment (12 weeks). Among PJIs managed with a one-stage exchange, 5 patients underwent a similar postoperative antibiotic treatment duration. Implant removal was performed in 6 cases with these patients adhering to a 6-week antibiotic regimen, as no implants were retained.

A suitable PO antibiotic regimen was found for 127 patients (98%). The median duration of IV antibiotic therapy for patients who switched to an oral antibiotic regimen was 7 days (IQR 7–7).

The oral and IV antibiotic regimens most frequently prescribed in the oral group were penicillins, accounting for 68% and 73% of cases, respectively. The second most used antibiotics were IV glycopeptides and PO macrolides/lincosamides (Table 2). In the 2 patients exclusively treated with IV antibiotics, cephalosporins and carbapenems were administered, respectively. 1 of these patients received adjunct oral antibiotics with quinolone. Rifampicin was used in only 1 case.

**Table 2:** Antibiotic treatment details: comparison of Herlev and RNOH oral subgroups. Values are count (%)

Item	Herlev	RNOH
	n = 127	n = 121
Intravenous antibiotic use		
Cephalosporins	20 (16)	0
Glycopeptide	27 (21)	0
Carbapenems	5 (3.9)	0
Penicillins	93 (73)	0
Other IV antibiotics	5 (3.9)	0
IV antibiotic regimes with adjunct		
PO antibiotics	2 (1.6)	NA
Oral antibiotic use		
Rifampicin	1 (0.8)	36 (30)
Quinolones	17 (13)	41 (34)
Penicillins	86 (68)	40 (33)
Tetracyclines	0	33 (27)
Macrolides/lincosamide	22 (17)	23 (19)
Other PO antibiotic	20 (16)	15 (12)
Combination IV antibiotic use		
Single antibiotic therapy	52 (41)	NA
Double antibiotic therapy	68 (54)	NA
Triple antibiotic therapy	7 (5.5)	NA
Quadruple antibiotic therapy	0	NA
Combination PO antibiotic use		
Single antibiotic therapy	67 (53)	58 (48)
Double antibiotic therapy	55 (43)	57 (47)
Triple antibiotic therapy	5 (3.9)	6 (5.0)
Quadruple antibiotic therapy	0	0

IV: intravenous, PO: oral, NA: not applicable, RNOH: Royal National Orthopaedic Hospital.

A change in the type of IV treatment was necessary in 43 patients following the postoperative regimen. Before transitioning to oral therapy 37 (29%) patients had 1 change in IV regimen, while 6 (4.7%) had 2 changes until the final antibiotic regimen was found. The median number of postoperative days until the final regimen was found was 3 days (IQR 2–5). The median LOS for patients who underwent at least 1 antibiotic change was 9 days (IQR 7–14), whereas patients who had no changes had a median LOS of 7 days (IQR 7–7).

Regarding the empirical IV antibiotic regimen, 90 (70%) among all patients were initially treated with a combination of penicillin G and cloxacillin. Of these cases, 62 patients (69%) completed their IV antibiotic treatment either with a combination of penicillin G and cloxacillin or single antibiotic therapy with 1 of these penicillins. 28 patients were postoperatively changed from the standard empirical regimen for the following reasons: perioperative sampling growth (22), positive blood cultures (1), suspicion of allergic reactions (1), concurrent infections (3), kidney failure (1). Initially administered IV antibiotics other than the standard empirical regimen were used in 39 patients for 1 or more of the following reasons: drug allergies (15), preoperative joint aspirations (5), preoperative sampling (9), wound swabs (3), preoperative blood cultures (3), monotherapy with penicillins only (3), concurrent infections (1), kidney failure (1), unexpected positive sampling postoperatively (1) and unknown (4).

Further analysis of PJIs shows the following distribution of IV antibiotics: 45% received penicillins, 41% received glycopeptides, 32% received cephalosporins, 5% received carbapenems. Single, double, and triple antibiotic therapy was given to 15, 5, and 2 patients, respectively.

### Microbiological findings

In 82 cases (64%) intraoperative tissue sampling was positive. The most frequently identified microbiological organism was *Staphylococcus aureus* in 21 patients (26%), the second being *Staphylococcus epidermidis* in 19 patients (23%). Multidrug-resistant bacteria were seen in 7 cases (8.5%) and managed with prolonged IV antibiotics before switching to an appropriate oral regimen. In 47 patients (36%) no bacteria were found. Patients with culture-negative infections who were eligible for oral treatment remained on an empirical antibiotic regimen unless an ADR prevented this or the patient had any prior history of allergic reactions or intolerance to penicillins.

### Primary outcome

Definite treatment failure occurred in 16 out of 124 patients overall (13%) and in 16 out of 122 patients (13%) treated with oral antibiotics (Table 3). All 16 patients with treatment failure underwent reoperation. The types of infections were osteomyelitis (12), PJIs (2), and septic arthritis (2). Among the 16 patients, 5 cases were culture negative. 2 patients could not complete a suitable oral regimen and had no failures. Clinical failure was identified at median of 11 (IQR 6–24) weeks postoperatively.

**Table 3:** Clinical outcome: comparison of Herlev, RNOH, and OVIVA oral subgroups. Values are count and percentage when specified

Item	Herlev	RNOH	OVIVA
	n = 127	n = 121	n = 509
Lost to follow-up	0	0	
Deaths	5	4	
Not curative intent	0	5	
Evaluable patients	122	112	509
Definite failures at 1 year, n (%)	16 (13)	16 (14)	67 (13)

RNOH: Royal National Orthopaedic Hospital, OVIVA: Oral Versus Intravenous Antibiotics.

### Secondary outcomes

68 ADRs (65 in the oral subgroup) occurred (Table 4). During the first 6 weeks of antibiotic treatment, 47 (36%) of all patients experienced an ADR. Gastrointestinal problems were the most common type of ADR, and biochemical/hematological was the second most common. Most ADRs were managed with close monitoring and/or symptomatic treatment. In the oral group, 8 patients (6.3%) required a change in antibiotics, and 1 patient was changed back to IV antibiotics due to ADRs. 2 patients (1.6%) in the oral group stopped their IV antibiotics before planned, but total length of antibiotic treatment was still 6 weeks, and no failures were observed in these 2 cases. Readmission rate due to ADRs was 4.7% (5 patients in the oral group and 1 patient in the IV group). In these 6 cases the readmission was due to gastrointestinal ADRs (1 of the patients in the IV group started with an oral regimen but was changed to IV because of gastrointestinal ADRs).

**Table 4:** Incidence of adverse drug reactions, their management, and readmissions: comparison of Herlev and RNOH oral subgroups. Values are count (%)

Item	Herlev	RNOH
Gastrointestinal		
Nausea/vomiting	21 (17)	22 (18)
Diarrhea	21 (17)	17 (14)
Reflux	4 (3.1)	2 (1.7)
Taste disturbances	2 (1.6)	1 (0.8)
Lethargy	1 (0.8)	0
Neuromuscular		
Dizziness	1 (0.8)	1 (0.8)
Headaches	0	1 (0.8)
Sweating	0	1 (0.8)
Muscle cramps	0	0
Unspecific (paresthesia)	1 (0.8)	NA
Biochemical/hematological		
Hypokalemia (< 3.5 mmol/L)	8 (6.3)	0
Creatine kinase (> 300 U/L)	0	0
Liver dysfunction (ALT > 100 U/L)	0	2 (1.7)
Kidney dysfunction	2 (1.6)	NA
Neutropenia (< 1,000 cells/µL)	0	2 (1.7)
Eosinophilia (> 500 cells/µL)	0	0
Thrombocytopenia (< 100x10^3^ and decrease > 50%)	0	0
Immunological		
Drug fever	1 (0.8)	0
Rash	1 (0.8)	8 (6.6)
Anaphylaxis	1 (0.8)	0
Eosinophilic pneumonia	0	0
“General discomfort”	1 (0.8)	NA
Microbiological		
Thrush	0	0
* Clostridium difficile* infection	0	0
Management of ADRs		
Closer monitoring/symptomatic treatment only	22 (17)	31 (26)
Dose change	1 (0.8)	4 (3.3)
Change in antibiotic/same route	8 (6.3)	15 (12)
Stopped early	2 (1.6)	6 (5.0)
Readmission during the first 6 weeks of treatment		
Readmissions due to ADRs	5 (3.9)	4 (2.2)
Readmission > 24 hours for any other cause	12 (9.4)	1 (0.5)

RNOH: Royal National Orthopaedic Hospital, NA: not applicable.

5 patients experienced line complications and all were mechanical. Total line complications/1,000 line-days was 4.4. The 2 patients in the IV group did not have any line complications. Line complications were all managed with replacement, and no cases of infection or thrombophlebitis were reported.

## Discussion

The aim of our study was to evaluate the clinical outcome, antibiotic treatment details, and complication rates based on treatment according to the OVIVA protocol. We found a comparably low failure rate of 13% among patients who were able to transition to oral antibiotics. This closely aligns with the results reported in both the OVIVA study (13%) and the RNOH study (14%) for their oral treatment group, again reiterating OVIVA’s reproducibility [7,8].

These results are particularly notable given the narrow-spectrum antibiotic regimen employed, which relied heavily on penicillins. There were no cases of oral tetracycline use and only 1 case of rifampicin use. Conversely, rifampicin was used in 26% of oral cases in the RNOH study and in 27% of all patients in the OVIVA study [7,8]. While certain literature reports the benefit of rifampicin use in PJIs [10-12], a recent randomized controlled trial found no significant advantage in adding rifampicin for early staphylococcal PJIs treated with DAIR [13].

The predominant use of penicillins in this study could be subject to criticism, particularly regarding their limited bone penetration [14,15]. Although direct data on antibiotic penetration into infected bone is lacking, the findings do not support this concern, as most surgically treated BJIs were managed primarily with gram-positive cover alone, yet clinical outcomes were comparable to those reported in the OVIVA and RNOH studies. As antibiotic bone penetration has not been investigated in clinically infected bone, the counterarguments against switching to oral antibiotics are not supported by data [15]. However, a porcine study has shown decreased penetration of cefuroxime into infected bone due to the destructive processes associated with osteomyelitis [16]. Furthermore, a retrospective study of surgically treated diabetic foot infections found no difference in outcomes when comparing the use of amoxicillin/clavulanate with other oral antibiotics [17]. The predominant use of penicillins and the high proportion of patients who successfully transitioned to oral therapy may also reflect the effectiveness of the surgical intervention, which has been described as a crucial step in the management of BJIs [18].

Numerous studies indicate the safe usage and effectiveness of oral antibiotics, including a systematic review of 25 prospective trials demonstrating no difference in clinical efficacy between patients receiving IV-only therapy and those switched to oral antibiotics after initial IV treatment, in both blood and bone infections [19]. In line with this study, Sendi et al. recommend that treatment stratification with oral antibiotics be guided by a multidisciplinary team, considering clinical status, source control, and oral antibiotic tolerance [15,19]. Additional benefits of using oral antibiotics compared with IV therapy include greater cost savings [9,20,21], shorter hospital stays [8,19], and fewer catheter-related complications [7,19].

### Similarities and differences in demographics and antibiotics

Although our patient cohort had more ASA ≥ 3 and fewer ASA 1 patients than the RNOH study, our study observed similar failure rates [8]. The difference in median days of antibiotic therapy may be attributed to the lower rate of PJIs compared with the RNOH study (17% vs 76%), as PJIs typically require longer antibiotic treatment [5].

One possible reason for the differences in antibiotic treatment could be variations in the resistance pattern of the microbiological findings. The RNOH study reported staphylococcal species as the most commonly identified organisms, similar to the findings in our study. The differing rates of patients switching to oral antibiotics between our study and the RNOH study (98% vs 66%, respectively) may further suggest differences in microbiological resistance patterns.

Another reason could be the difference in the rate of culture-negative infections, with our study having a higher proportion. This might be due to differences in microbiology assessments/analysis and strategies. Because bacteria are known to invade the small canalicular network of bones, the high number of culture-negative infections could be a result of imprecise sampling or preoperative suppressive antibiotic treatment [22]. However, a meta-analysis of the literature did not find a significant difference in bacterial yield based on antibiotic use prior to bone biopsy in nonvertebral osteomyelitis [23]. The authors believe the risk of preoperative suppressive antibiotics is low because it is standard practice to cease antibiotic use at least 14 days prior to surgery. Thus, it seems unlikely to account for all the culture-negative cases observed. The RNOH study reported a culture-negative infection rate of 29% in the IV subgroup and 22% in the oral subgroup [8], whereas the rate was 36% in our study. When comparing with neighboring Scandinavian countries, a study from Norway reported that 22% of 76 patients with confirmatory signs of fracture-related ankle infection had negative cultures [24], while a study with Swedish data found that 20% of 202 patients with PJIs of the knee had culture-negative infection [25]. The diagnostic accuracy in this study might have been improved by the inclusion of histopathological analysis, as recommended in other studies [26,27].

Regarding secondary outcomes, the findings of the RNOH study indicate a tradeoff between longer LOS with IV treatment and a higher incidence of gastrointestinal ADRs with oral treatment. Similarly, the most reported ADR was gastrointestinal.

### Limitations

This study was limited by its retrospective nature, which could underestimate the amount of complications/ADRs and clinical outcome. The BJIs reported in this study represented a heterogeneous group with different types of infections. Osteomyelitis was by far the most frequent type of BJI, whereas the relatively low number of other infections could represent a limiting factor. This study did not include histological analysis to assess definite failure, as it is not routinely performed by the department of pathology. Furthermore, this study did not include specifics on which criteria for treatment failure were met in each case, limiting the analysis of this subgroup. Data regarding preoperative suppressive use of antibiotics was not included, which might have influenced the number of culture-negative infections in our study.

A pre-implementation control group was not included; therefore, outcomes were assessed in comparison with the findings of the OVIVA trial and the RNOH study. This study is susceptible to selection bias due to a preference for an oral treatment strategy, with IV antibiotics reserved only for cases where no appropriate oral option is available, and, furthermore, only patients treated at Herlev Hospital were included. Finally, comparison of antibiotic type and duration may be limited by differences in treatment approach between the OVIVA and our study.

### Conclusion

We found a comparably low failure rate of 13% among patients who were able to transition to oral antibiotics when applying the treatment protocol from the OVIVA study. This closely aligns with the results reported in both the OVIVA study (13%) and the RNOH study (14%) for their oral treatment group, again reiterating OVIVA’s reproducibility.

*In perspective,* the findings reinforce the viability of oral antibiotics as an alternative to prolonged IV therapy and provide a framework for global comparisons of antibiotic strategies.

## Notes

Author contributions were assigned according to the CRediT.RB: methodology, data curation, formal analysis, visualization, writing—original draft, writing—review and editing. AP: project administration, supervision, writing—review and editing. JB: conceptualization, methodology, data curation, writing—review and editing. HG: conceptualization, supervision, writing—review and editing

The authors would like to acknowledge the staff, surgeons, and their colleagues from the microbiology department for their contribution to the care and treatment of the patients with BJIs.

Handling co-editors: Marianne Westberg and Robin Christensen

*Acta* thanks Kristian Pilskog and Matthew Scarborough for help with peer review of this manuscript.
